# Impact of occlusal contact pattern on dental stability and oromandibular system after orthodontic tooth movement in rats

**DOI:** 10.1038/s41598-023-46668-x

**Published:** 2023-12-14

**Authors:** Menglin Wang, Jing Wang, Xiang Jin, Dedi Liu, Huan Bian, Yantao Zhao, Yanfeng Li

**Affiliations:** 1 Department of Stomatology, The Fourth Medical Center, Chinese PLA General Hospital, No. 51 Fucheng Road, Haidian District, Beijing, 100048 China; 2 Department of Stomatology, Beijing Chaoyang Hospital, Capital Medical University, Beijing, 100043 China; 3 Department of Orthopedics, The Fourth Medical Center, Chinese PLA General Hospital, Beijing, 100048 China; 4 Beijing Engineering Research Center of Orthopedics Implants, Beijing, 100048 China; 5 State Key Laboratory of Military Stomatology, Xi’an, 710032 China

**Keywords:** Biological techniques, Plant sciences

## Abstract

How to ensure dental stability in new positions and reduce the likelihood of relapse is a major clinical concern in the orthodontic field. Occlusal contacts between arches may affect the transmission of masticatory forces, thereby influencing the biological response of the periodontal and the oromandibular system. Occlusion factors that may influence the stability after orthodontic tooth movement (OTM) remain largely unknown. Hence, this research was conducted in order to investigate the influence of different occlusal contact patterns on tooth stability and oromandibular system including the masseter muscle and the temporomandibular joint following OTM. By modifying the occlusal surfaces, in vivo animal study models with distinct occlusal patterns corresponding to clinical circumstances were established. The relapse distance of teeth and the level of inflammatory factors in the gingival cervical fluid were analyzed. We also closely observed the histological remodeling of periodontal tissue, masseter tissue, and joint tissue after one week of relapse. Moreover, genes expression in the alveolar bone was analyzed to illustrate the potential biological mechanisms of relapse under the influence of different occlusal contact patterns following OTM. Different occlusal contact patterns after OTM in rats were established. The intercuspation contact between cusp and fossa group exhibited the lowest level of relapse movement, inflammatory factors and osteoclast activity (*P* < 0.05). On the other hand, groups with interferences or inadequate contacts exhibited more relapse movement, and tend to promote inflammation of periodontal tissue and activate bone resorption (*P* < 0.05). Adequate occlusal contacts without interference may enhance tooth stability and reduce the likelihood of relapse. After active orthodontic treatment, necessary occlusal adjustment should be made to achieve the desired intercuspation contact relationship and ensure adequate contact between the arches. The elimination of occlusal interferences is crucial to achieving optimal stability and promoting overall healthy condition of the oromandibular system.

## Introduction

Orthodontic Relapse is a common condition where the teeth tend to move back to their original position after the removal of orthodontic appliances^1^. This is a complex process influenced by numerous unpredictable and uncertain factors. According to surveys, the frequency of relapse after completion of orthodontic treatment ranges from 14 to 70%^2,3^. During orthodontic tooth movement (OTM), mechanical stresses are produced and stored in period ontium and gingival fibers, resulting in compressive and tensile stresses on the periodontal tissues of the stressed teeth^4^. These stresses are released after the removal of the orthodontic appliance, and relapse occurs as the teeth move in the initial direction, which is mediated by the osteoclastic activity of bone resorption^5^. Besides, various force-related occlusal factors, soft tissue factors, arch length limitations, and some physiological factors may also influence the effectiveness of treatment and the stability of outcomes^6^.

Ensuring stable tooth position after OTM is a key issue in orthodontics. In recent years, biologically modulating the bone remodeling process has become a major focus in research to increase stability after OTM. Various approaches have been explored to reduce relapsing tooth movement after orthodontic treatment, mainly through enhancing new bone production and inhibiting osteoclast activity^7^. There include the use of bisphosphonates, statins^8,9^, relaxin^10^, osteointegrin^11^, hormonal agents^12^, and low-energy lasers to regulate the bone remodeling process. These approaches work by regulating the release of cellular inflammatory factors, modulating the RANK/RANKL/OPG pathway to inhibit osteoclast differentiation and maturation, or increasing osteogenic activity by upregulating the expression of osteogenic genes such as BMP-2 and Runx2 and thus inhibits relapse. However, their effectiveness in inhibiting post-orthodontic relapse in humans is not yet clear, and further research is needed to establish their effects on local and systemic homeostasis, as well as their safety for clinical use.

Most experts agree that achieving high-quality orthodontic results leads to lower rates of post-orthodontic relapse. Nett et al. found that patients with high-quality endings had better occlusal relationship scores during the retention period compared to those without^13^. Littlewood et al. concluded that cusp-fossa intercuspation occlusal contacts promote orthodontic retention stability, which supports the idea of retention through occlusion, but there is a lack of experimental evidence^6^. Accurately and precisely adjusting the occlusal contact relationship is a crucial focus of the final stage of comprehensive orthodontic treatment^14^. Cusps-fossae occlusal contact patterns allow for smooth gliding of the mandibular cusps along the the maxillary cusps during movement, transmitting forces along the axis. Appropriate occlusal force stimulation is essential for the development of alveolar bone, maintenance of bone volume, and balance of bone transformation^15^. However, non-axial occlusal forces such as lateral or horizontal forces, torques, and rotational forces resulting from abnormal tooth contact can lead to the widening of the periodontal membrane gap, increased fiber tension within the periodontium, and in severe cases, periodontal membrane tearing and alveolar bone resorption, resulting in alterations ingingival cervical fluid (GCF) flow rate and composition^16^, upregulation of RANKL expression^17^, and increased pathological tooth mobility^18^.Occlusal contact patterns can stimulate changes in the periodontal tissues and subsequently affect tooth position stability, while the masticatory muscle system may produce different behavioral effects to compensate for the occlusal imbalance^19^. Molars occlusion also plays an important role in the mechanical environment of the internal structures of the temporomandibular joint (TMJ)^20,21^, affecting the position of the mandibular and the transduction of occlusal force between the upper and lower arch.

Occlusal contact refers to the three-dimensional contact relationships between the upper and lower teeth, including correspondence and contact relationships between the cusp fossae of the occlusal surfaces. Intercuspation is an occlusal contact pattern in which the cusp fossae between the maxillary and mandibular teeth intersect to achieve the most extensive and intimate contact^22^ Establishing maximal intercuspation is a crucial occlusal goal of orthodontic treatment^23,24^. However, during clinical orthodontic treatment, absolute parallel movement of the teeth is challenging, and necessary oblique movement occurs, making it difficult to achieve optimal cusp-fossa intercuspationocclusal contact^25^. The current objective of orthodontic treatment is to establish an optimal occlusion that is close to the ideal dentition in terms of function and aesthetics. However, little is known about the correlation between detailed occlusal factors and the stability of outcomes after orthodontic treatment^26^.

There is a lack of in-depth studies on the histological changes during relapse and the influence of occlusal contact on the stability of orthodontic outcomes^27^. Herein, the purpose of this study was to investigate the influence of different occlusal contact patterns of molar teeth on tooth stability and the oromandibular system, including the masseter and TMJ tissue. The goal was to illustrate the biological mechanisms of relapse after orthodontic treatment and provide experimental evidence for a more specific occlusal treatment objective that promotes post-orthodontic stability. Animal study models with distinct occlusal patterns were developed for this purpose.

## Methods

### Experimental design

**Figure 1:**
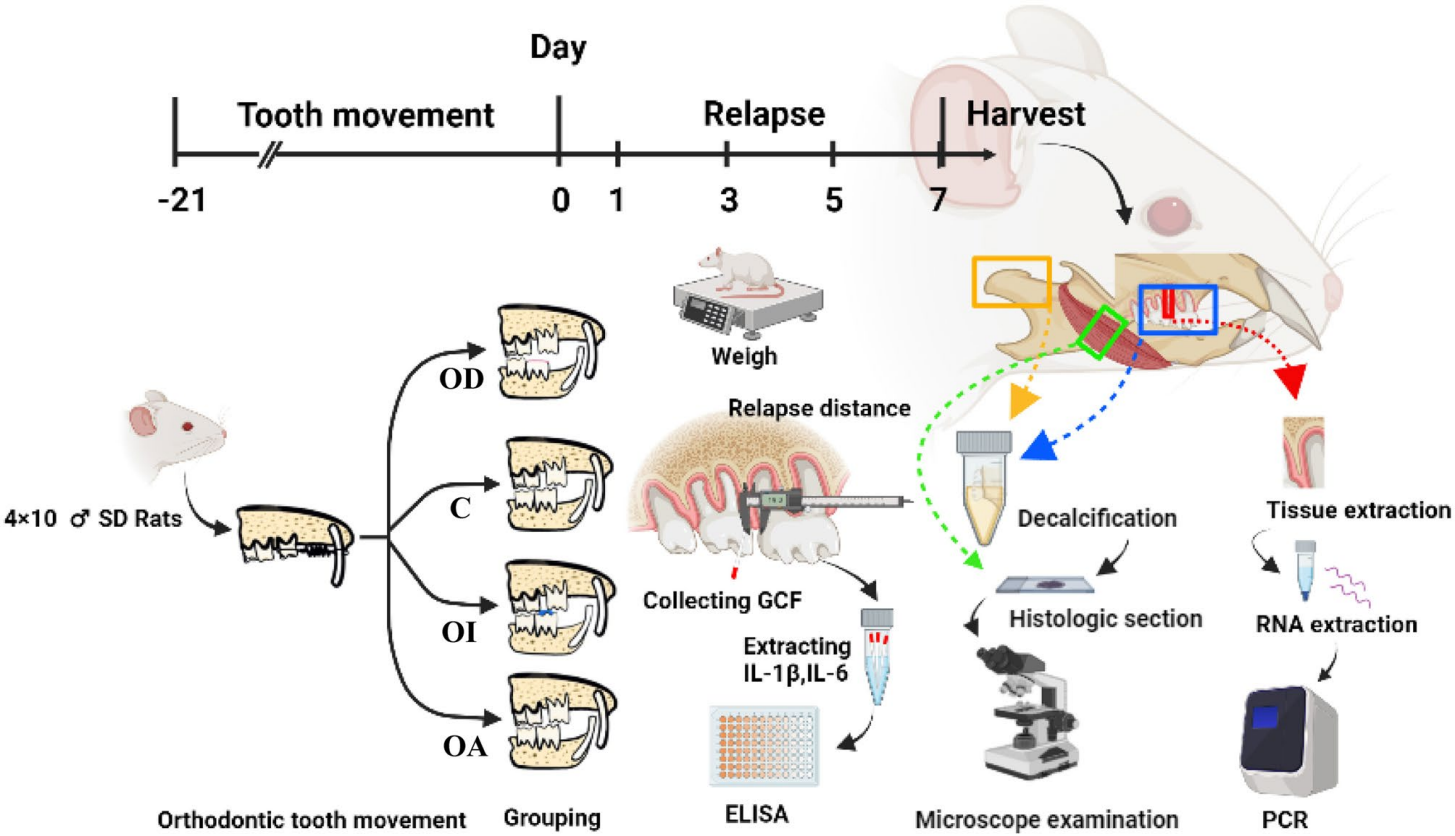
Experimental design. 40 male SD rats were randomly allocated into 4 groups with different established occlusal contact patterns after active orthodontic tooth movement of UM1. The force appliances were removed and the relapse distance, the general conditions, and the GCF were examined on day 1, 3, 5, and 7 of relapse. The left maxilla, masseter, and temporomandibular joint tissue were collected after the rats were sacrificed on day 7 for histological examinations and PCR.

The study was approved by the Ethical Committee on animal experimentation at the medical school of PLA General Hospital (Approval Number: ZJU20200455). Animals were acclimatized for at least one week and housed under regular laboratory conditions, with free access to standard laboratory feed and water prior to the experiment. Forty male Sprague–Dawley (SD) rats (aged 8 weeks, 300 ± 20 g) were randomly allocated into four groups based on the different designed occlusal contact patterns. 1: disocclusion (OD) group with the disconnected upper first molar (UM1) and lower first molar (LM1); 2: control (C) group with no intervention on the orthodontic tooth; 3: occlusal interference (OI) group with established occlusal interference between UM1 and LM1; 4: occlusal adjustment (OA) group with the established intercuspation relationship without interferences between UM1 and LM1. All animal experimental procedures were conducted in accordance with Animal Research: Reporting of In Vivo Experiments (ARRIVE) guidelines and were carried out under the National Institutes of Health guide for the care and use of laboratory animals. The experimental procedure was depicted in Fig. 1.

### Establishment of orthodontic tooth relapse model

**Figure 2:**
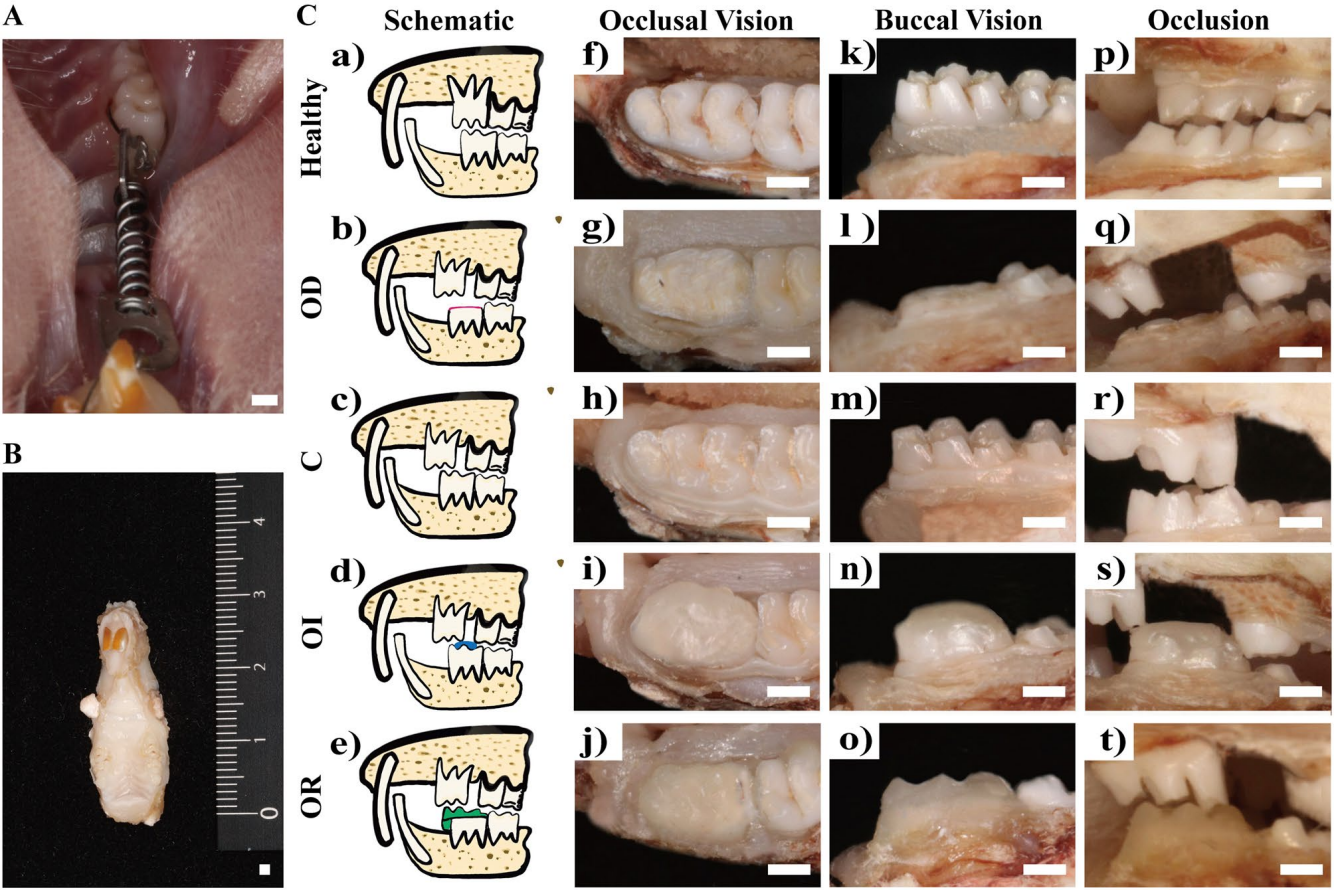
The Establishment of orthodontic tooth movement model (**A**). A closed spring was ligated between the left UM1 and the incisors. The samples of the left maxilla were collected subsequent to scarification (**B**). Different occlusal contact patterns were established once the force appliances were removed by the modification of the occlusal surface of the left LM1 (**C**). The schematic illusion of different occlusal contact patterns was shown in Ca–e, the occlusal vision of the left LM1 was shown in Cf–j, and the buccal vision was shown in Ck–o. Different occlusal patterns shown in Cp–t represented the static occlusal status during relapse.

The rats were anesthetized by intraperitoneal injection of 10% ketamine hydrochloride (0.08 ml per 100 g of body weight) and 2% xylazine hydrochloride (0.04 ml per 100 g of body weight). The cervical of the incisor and UM1 on the left half maxillary were ligated using a 0.2-mm ligature wire (CrNi, Morelli, Brazil), acid etched the tooth surface using 35% phosphoric acid (Gluma® Etch 35%, HeraeusKulzer, German), rinsed, blown dry, insolated, coated with the adhesive (Transbond, 3 M Unitek, Monrovia, Calif), and covered with a thin layer of resin (Filtek Flow composite, 3 M/ESPE, St.Paul, MN, USA) at the surface of the wire and the notch to prevent dislocation of the ligating wire and secondary sensitivity of the tooth. A closed coil nickel-titanium spring (Light, Sentalloy®, GAC, Dentsply) was ligated between the left maxillary incisor and UM1 for traction **(**Fig. 2A), and a force value was determined using a dynamometer (YS-31D, YDM, Japan) to assure a constant force release of 50 g. The force-activated device was checked daily. Springs were removed after three weeks of orthodontic traction to allow the UM1 to relapse after OTM for one week.

### Establishment of different occlusal contact patterns model

Different occlusal patterns were established once the springs were removed by modification on the occlusal surface of the left LM1. The surfaces of the left UM1 and LM1 were cleaned using alcohol cotton balls to remove surface food debris and soft scale. In the OD group, the height of the occlusal surface of LM1was lowered by approximately 1 mm using a dental diamond bur (TF-22, MANI, Japan). Visual inspection combined with the folded occlusal paper examination was applied to confirm that the left LM1 and UM1 were out of occlusal contact (Fig. 2Cb, g, l, q). In the OI group, the orthodontic stainless-steel wire (0.0198 × 0.025, 3 M Unitek, USA) combined with resin (Filtek P60, 3 M, USA) was bonded to the occlusal surface of LM1 to create a 1.0-mm height occlusal interference (Fig. 2Cd, i, n, s). The height of the occlusal interference was measured with the use of a digital vernier caliper (111–101, Sanliang, Japan) to achieve experimentally designed height. To verify the establishment of interference contact, the rats were subjected to executing both central and marginal movements.

In the OA group, after removing the spring, the movement distance of UM1 was measured as D mm. The impression of the occlusal surface of LM1 was performed using silicone rubber (Silagum, DMG, USA). The height of the occlusal surface LM1 was lowered by 1 mm. Afterwards, the original vertical height of the LM1 and the morphology of the occlusal surface were restored using flowable resin (Filtek Flow composite, 3 M/ESPE, St.Paul, MN, USA) with the aid of the former impression, with the edge of the mesially extended surface by D mm serving as the new edge for the reconstruction of the occlusal surface. In brief, through modification on the occlusal surface of LM1, the original intercuspation occlusal contact pattern between LM1 and the UM1 was reestablished in the new position after the UM1 was mesially moved (Fig. 2Ce, j, o, t). The studies also performed direct visual inspection combined with occlusal paper examination and necessary occlusal adjustment to verify the formation of a non-interfering occlusal contact pattern during dynamic movement of the mandibular.

### Measurement of relapse distance and general condition

The daily behavioral performance of the rats was closely observed and recorded. On day 1, 3, 5, and 7 following the removal of the force appliances, the body weight (g) was recorded. The studiers monitored intraoral occlusion model to verify its proper maintained status, and any dislodgement or damage should be repaired promptly. Samples were reincorporated in case of animal death or model failure.

**Figure 3:**
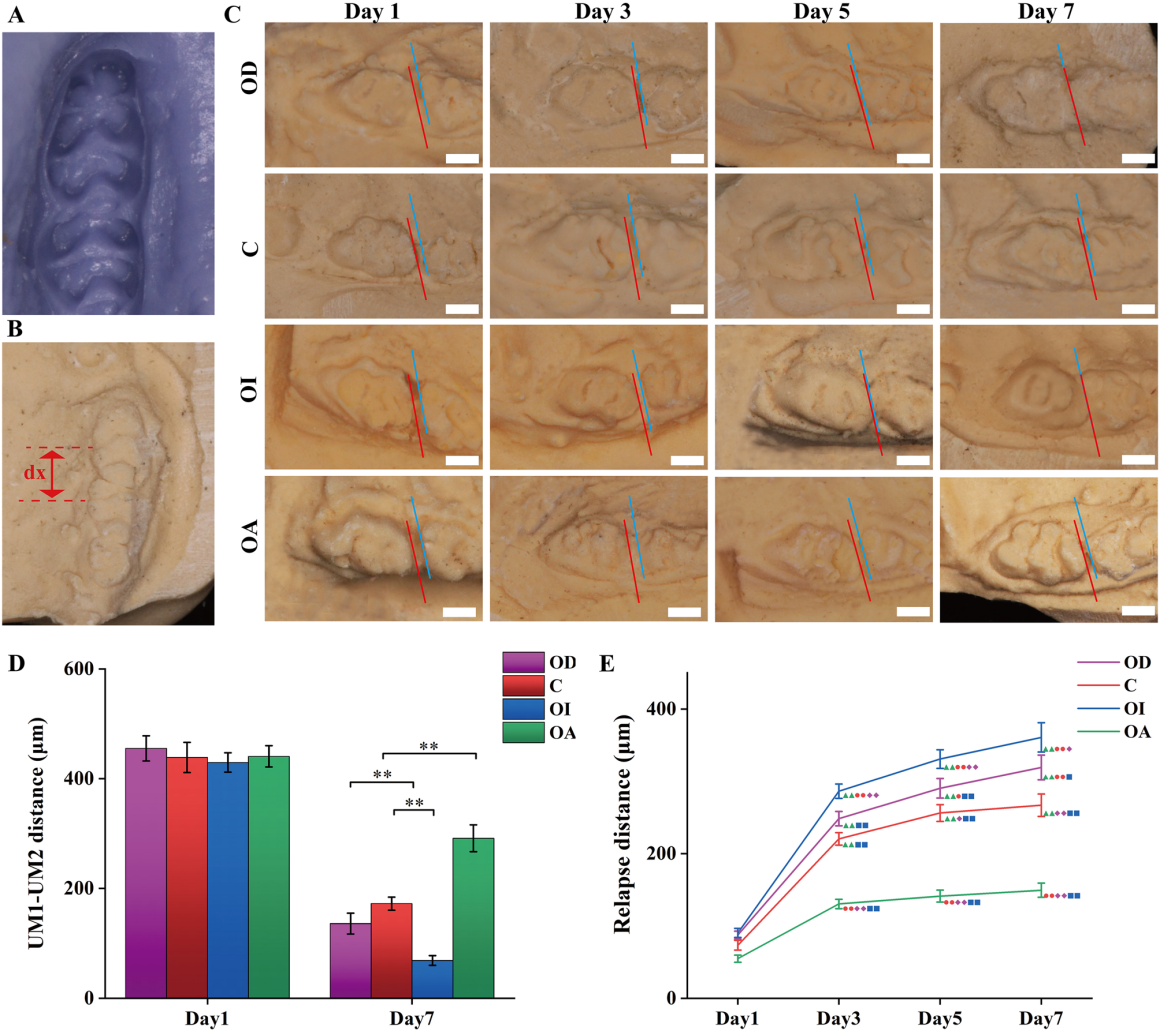
Measurement of the relapse distance of the left UM1 with different occlusal contact patterns after orthodontic movement. The relapse distance of the UM1 was measured by taking silicone impressions on day 1, 3, 5 and 7 (**A**). The distance between the palatal groove of UM1 and UM2 at the gingival level was recorded as dx (**B**). The relapse distance of UM1 was measured on the model (**C**, **D**).The blue and red lines represented the margins of UM1 and UM2. Scale bar = 1.0 mm. The relapse distance of different groups was shown in (**E**), with the gradient of the connected lines showing the rate of relapse. △ represented statistical difference existed between OA group and* P* < 0.05, △△, *P* < 0.01. ○ represented statistical difference existed between C group and* P* < 0.05, ○○, *P* < 0.01; ◊ represented statistical difference existed between OD group and *P* < 0.05, ◊◊, *P* < 0.01; □ represented statistical difference existed between OI group and *P* < 0.05, □□, *P* < 0.01.

To measure the relapse distance, the anesthetized animals were fixed supine on the experimental table with their maxilla parallel to the ground. The impressions of the left maxilla were taken with customized trays and silicone impressions (Fig. 3A), and the plaster model was perfused. Models were trimmed to ensure that the occlusal surface of the UM1 was parallel to the ground, and the distance between the palatal groove of UM1 and the UM2 at the gingival level was measured on the model and recorded as dx (Fig. 3B). The distance that was measured immediately after removing orthodontic appliances was recorded as d0, and the relapse distance was calculated as d = d0−dx.

### Examination of IL-1β and IL-6 expression in GCF

GCF samples were collected and prepared as described in the research of Lamsteret al.^28^ on day 1, 3, 5, and 7 of relapse. In brief, the cervical of left UM1 was cleaned, isolated, and gently air-dried. The GCF was collected from the lingual and labial cervical groove with a sterile paper strip (PerioPaper, Proflow, USA). The paper strip was inserted into the gingival sulcus without damaging the periodontal tissue or causing bleeding, held for 30 s to capture the GCF, and then transferred to a weighed Eppendorf tube. The tube was added with 100 μl PBS solution, and shaken for 1 h at room temperature. The supernatant was extracted after 10 min of 1000-rpm centrifugation at 4 °C. The enzyme-linked immunosorbent assay (ELISA) kits (ab255730/ab234570, Abcam, UK) were utilized for IL-1β and IL-6 quantification according to the manufacturer’s instructions. The concentrations of IL-1β and IL-6 were calculated using the standard curve.

### Examination of bone remodeling-related mRNA expression

Three rats were randomly selected from each group after being sacrificed.And the left maxilla was collected. Cortical and cancellous bone tissue was obtained within 1-mm distal to the middle third of the distal root of UM1. Total RNA was extracted and complementary DNA was prepared from total RNA using the reverse transcription kit (Takara Bio, Shiga, Japan). A quantitative real-time polymerase chain (qRT-PCR) reaction was performed. The primer sequences were as follows: BMP-2 forward, 5’-TCTTCCGGGAACAGATACAGG-3’; BMP-2 reve-rse, 5’ -TGGTGTCCAATAGTCTGGTCA-3’; Runx2 forward, 5’-GACTGTGGTTACCGTCATGGC-3’; Runx2 reverse, 5’-ACTTGGTTTTTCATATCAGCGGA-3’; OPG forward, 5’-CCTTGCCCTGACCACTCTTAT-3’; OPG reverse, 5’-CACACACTCGGTTGTGGGT-3’; RANKL forward, 5’-AGCCGAGACTACGGCAAGTA-3’; RANKL reverse, 5’-AAAGTACAGGAACAGAGCGATG-3’; GAPDH forward, 5’ AGGTCGGTGTGAACGGATTTG-3’; GAPDH reverse, 5’-GGGGTCGTTGATGGCAACA-3’. Relative gene expression was quantified using the equation of gene expression = 2−(△Ct Sample−△Ct Control) 42.

### Histological analysis

The left maxilla (Fig. 2B) and left joint tissue of each sample were fixed in 4% paraformaldehyde at pH 7.2 for 48 h and decalcified in 10% EDTA solution (ethylenediaminetetraacetic acid 10%, 0.5 M) for 4–6 weeks, and then embedded in paraffin for subsequent histological sectioning. Sections of the maxilla were cut along the long axis of the tooth in a mesiodistal direction. During the sectioning of the joint, the lateral margin of the zygomatic arch was kept parallel to the bottom of the wax block, and sagittal sections were made in a continuous manner. Serial parasagittal sections for 5 μm were mounted on slides. We stained the selected sections of the maxilla using hematoxylin–eosin (HE) staining, Masson staining, and tartrate-resistant acid phosphatase (TRAP) staining. Sections containing tissue near the median sagittal position of the joint sample were selected for HE staining and toluidine blue staining. Left masseter tissue was taken, fixed, dehydrated and paraffin-embedded, serially sectioned along the cross-section of the tissue. The morphologically intact sections of the masseter were selected for HE staining. Stained sections were examined under the microscope (CTR6500, Leica, German), and digital images were collected and processed with the automatic analysis system (QWin Plus, Leica, German). Cell counting and bone histomorphometry analysis were performed with the application of the Image J (NIH, Bethesda, USA) software.

### Statistical analysis

The normality of the collected data was examined, and the experimental data were presented as mean ± standard deviation. All statistical analyses were performed using the SPSS (version 17.0, IBM, Chicago, IL) software. The one-way ANOVA analysis accompanied by the least squares difference test was utilized to compare the results of distinct groups. Significance was considered at *P* < 0.05.

### Ethics approval and consent to participate

The study was approved by the Ethical Committee on animal experimentation at the medical school of PLA General Hospital (Approval Number: ZJU20200455). All methods were carried out in accordance with relevant guidelines and regulations. All applicable international, national, and/or institutional guidelines for the care and use of animals were followed.

## Results

### Effect of occlusal contact patterns on the general condition

**Figure 4:**
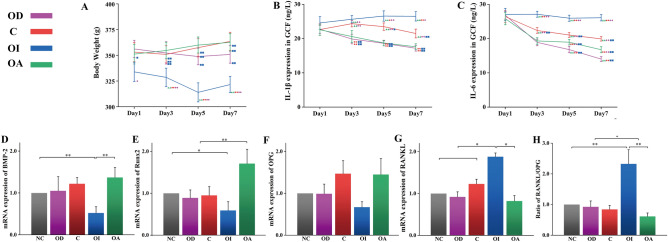
The weight changes of rats with different occlusal patterns (**A**). Influence of occlusal contact patterns on IL-1β (**B**) and IL-6 (**C**) expressions in GCF. △represented statistical difference existed between OA group and* P* < 0.05, △△, *P* < 0.01.○ represented statistical difference existed between C group and* P* < 0.05, ○○, *P* < 0.01; ◊ represented statistical difference existed between OD group and *P* < 0.05, ◊◊, *P* < 0.01; □ represented statistical difference existed between OI group and *P* < 0.05, □□, *P* < 0.01. Influence of different occlusal patterns on mRNA expression of bone remodeling-related genes, including osteogenesis genes BMP-2 (**D**), Runx2 (**E**), and OPG (**F**); and osteoclast gene RANKL (**G**). The ratio of RANKL/OPG was shown in (**H**). *, *P* < 0.05. **,* P* < 0.01.

All established occlusal patterns were well maintained under monitoring during relapse. Samples in the OI group initially ate less and chewed faster after bonding the resin interference on the occlusal surface of LM1, and exhibited the behavioral response of facial grooming. The body weight in the OI group tended to decrease and was significantly less than other groups (*P* < 0.05) as shown in Fig. 4A. The weight of the OD group decreased at the beginning and rebounded at day 7. No significant difference was detected in body weight among the OD, C, and OA groups (*P* > 0.05).

### Effect of occlusal contact patterns on relapsing distance

After 21 days of orthodontic traction, all samples produced significant movement of the left UM1 (Fig. 3D), with a mean distance of 440.17 ± 22.38 μm. No difference was observed among different groups (*P* > 0.05). On day 7, all four groups with established occlusal contact patterns showed different degrees of relapse (*P* < 0.05). The most severe relapse was observed in the OI group, and the least relapse in the OA group with a minor relapse distance and relapse rate (Fig. 3C-E) (*P* < 0.05). Compared to the C group, the OD group showed longer relapse distance (*P* < 0.05). The rate of tooth relapse in each group exhibited a progression from faster to slower (Fig. 3E).

### Effect of occlusal contact patterns on IL-1β and IL-6 expression in GCF

The overall trend changes showed a continuous decrease of IL-1β in the OA and OD groups, an increase in the OI group, andan increase followed by a decrease in the C group (Fig. 4B). IL-6 decreased continuously in all four groups (Fig. 4C). Both factors were most prevalent in the OI group (*P* < 0.05). IL-6 expression was the lowest in the OA group, and IL-1β expression was the lowest in the OA and OD groups compared to the C group (*P* < 0.05).

### Effect of occlusal contact patterns on bone remodeling-related mRNA expression

The expression of osteogenic genes BMP-2 (Fig. 4D) and Runx2 (Fig. 4E) was significantly lower in the OI group (*P* < 0.05), whereas RANKL (Fig. 4G) was highly expressed compared to other groups. The OA group had substantially higher Runx2 levels than other groups (*P* < 0.05). The RANKL/OPG ratio (Fig. 4H) was highest in the OI group and the lowest in the OA group (*P* < 0.05), indicating that active osteoclastic activity occurred in the OI group. No significant difference was detected between the OD and C groups (*P* > 0.05). Moreover, the expression of OPG did not differ among groups (Fig. 4F).

### Effect of occlusal contact patterns on periodontal tissue remodeling

**Figure 5:**
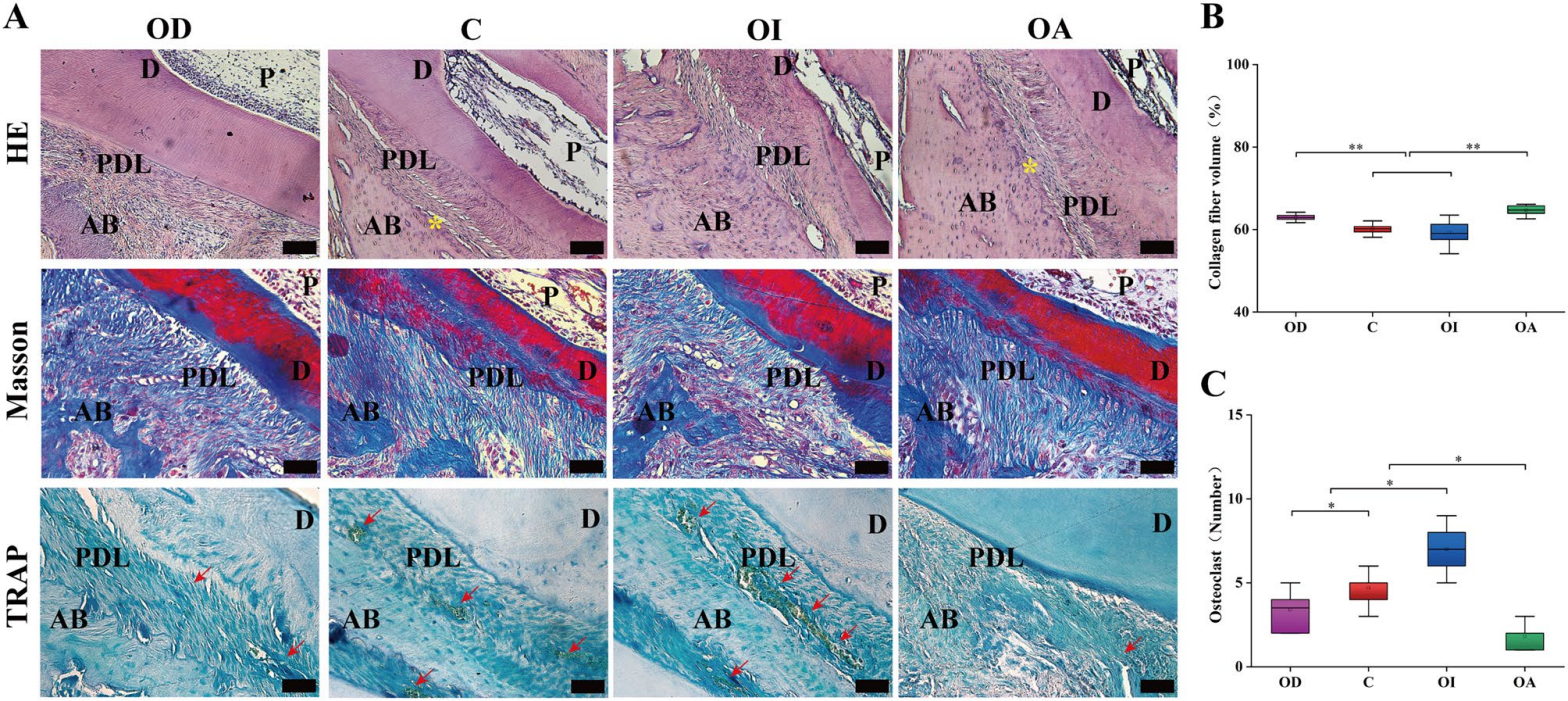
The periodontal tissue remodeling during relapse (**A**) with different occlusal patterns in ×20 magnification on HE-stained, Masson-stained, and TRAP-stained sections. P, dental pulp, D, dentin, PDL, periodontal ligament, AB, alveolar bone, *, new bone,  → , osteoclast appeared as trap-stained brown-positive multinucleated giant cells. Scale bar = 100 μm. Quantitative analysis results of collagen fiber volume (**B**) and osteoclast number (**C**) on tissue sections, data were presented as mean and standard deviation. *, *P* < 0.05. **, *P* < 0.01.

The remodeling of periodontal tissue was observed on the distal side of the teeth, which was the tension side during tooth movement and the pressure side during relapse. The periodontal fibers in the OD group were contracted and bent. The periodontal fibers in the C group were stretched with a few fractures, and new bone was seen; the periodontal fibers in the OI group were disorganized, and many fractures were found. In the OA group, the periodontal fibers were stretched and arranged more systematically, with no fracture. We observed more new bone on the distal side of the root (Fig. 5A). Based on the histomorphometry analysis of Masson-stained sections (Fig. 5B), collagen fiber volume (CFV) in the OA and OD groups was evidently greater than that in the C and OI groups (*P* < 0.01). There were no statistical differences between the OA and OD groups and between the C and OI groups (*P* > 0.05).The number of osteoclasts (Fig. 5C) was the lowest in the OA group, the highest in the OI group, and the number of osteoclasts in the C group was greater compared to the OD group, with statistical differences between each groups (*P* < 0.05).

### Effect of occlusal contact patterns on masseter tissue

**Figure 6:**
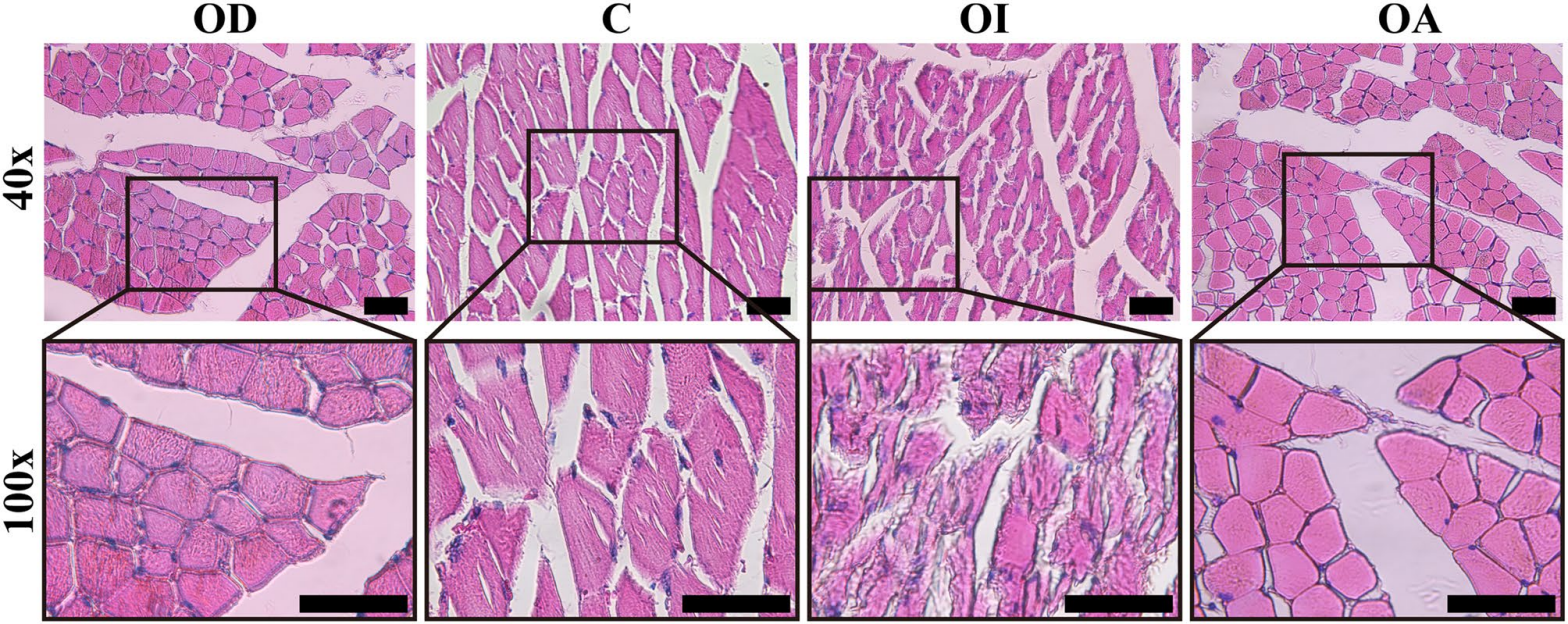
The influence of different occlusal patterns on the masseter tissues, HE-stained in 40× and 100× magnification.Scale bar = 50 μm.

The masseter tissue (Fig. 6) in the OD group was healthy, the cross-section of muscle fibers was round or polygonal, and the nucleus was located at the edge. The morphology in the OA group was similar to that of the OD group. A limited number of muscle fiber discontinuities were observed in the masseter tissue of the C group. In the OI group, the arrangement of muscle fibers was relatively disorganized, and the damaged changes were observed, alongside a loose arrangement of myogenic fibers, more pronounced fiber fractures, and lysis-like changes. Vascular congestion and inflammatory cell infiltration were found in some interstitial spaces.

### Effect of occlusal contact patterns on temporomandibular joint tissue

**Figure 7:**
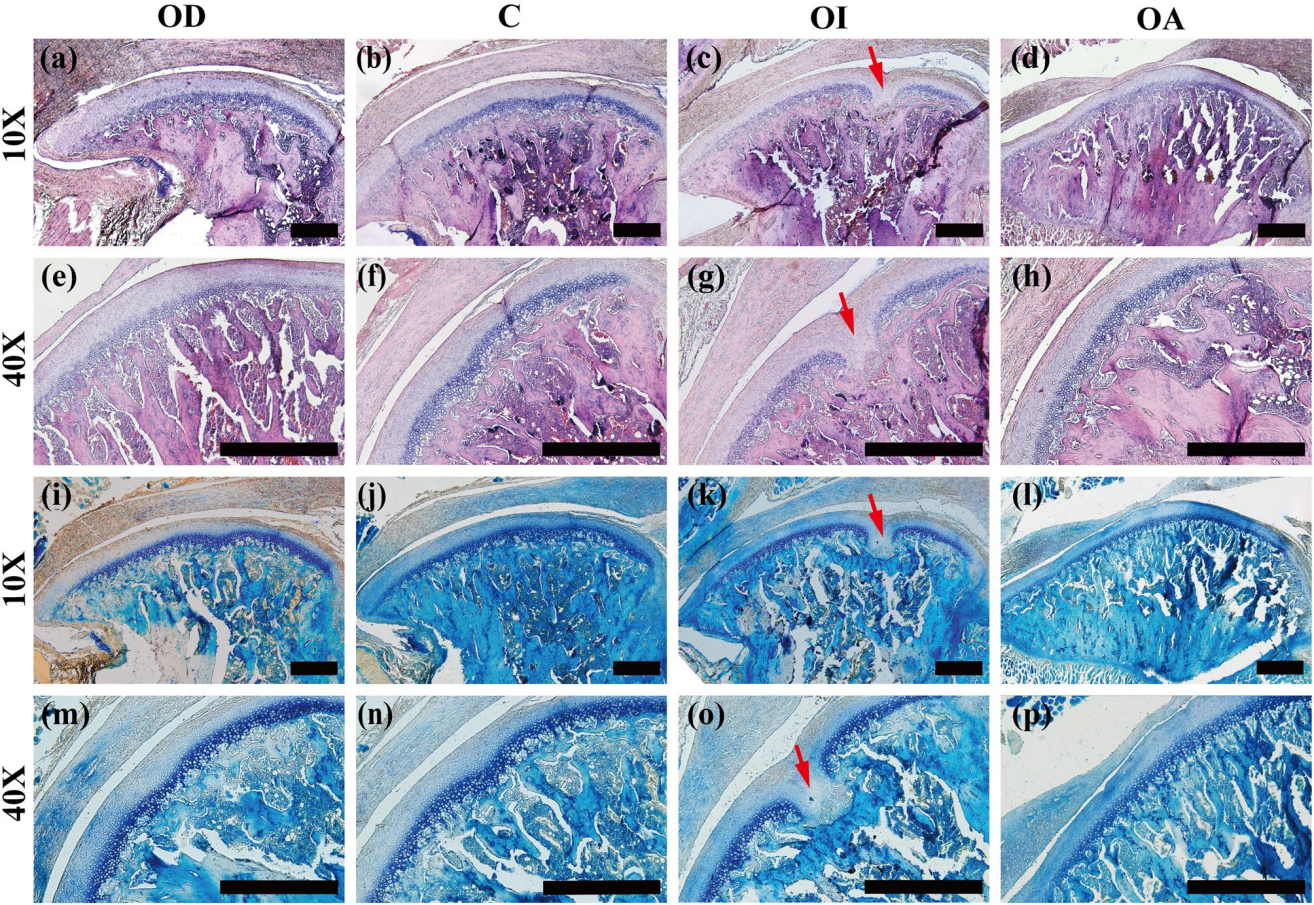
Histological sagittal sections of central TMJ condylar cartilage, HE-stained (**a**–**h**) and toluidine blue-stained (**i**–**p**) in 10× and 40× magnification. → represented the proliferation of condylar cartilage. Scale bar = 200 μm.

The matrix in the deep proliferative layer and the hypertrophic layer of TMJ sections from the OD, C, and OA groups was uniformly deep purplish-red. Proliferative changes in the cartilage were observed in the joint sections of the OI group, with cartilage projections that penetrated the central condylar cartilage and the subchondral bone of the femur (Fig. 7). In close proximity to the proliferative zone, localized, uniform eosinophilia and proteoglycan loss was observed.

## Discussion

Orthodontic treatment involves the reconstruction of occlusion, which is achieved in a shorter time compared to the natural process of establishing a stable cusp-fossa intercuspationocclusal pattern that is through long-term grinding and coordination of the maxillary and mandibular teeth. Therefore, occlusal adjustments are necessary to simulate the physiological wear and achieve a more stable occlusion^15^. The occlusal contact patterns established in the current study corresponded to actual clinical circumstances, indicating different occlusal contact statuses after OTM. This research provides clinical value by exploring how different occlusal contact patterns influence biological remodeling and the health of the oromandibular system, which can aid in achieving the goals of orthodontic treatment and occlusaladjustment.In the cusp-fossa intercuspationocclusal contact pattern, the occlusal forces are effectively decomposed by the cusp-fossa anastomosis at appropriate values, which is a healthy stimulus for the periodontal tissues.

The first molars of rats share structural and functional similarities with human molars, including being multi-rooted teeth with prominent cusps and clear sulcus patterns on the occlusal surface, and there is an intercuspation occlusion between the upper and lower molars^29^. Meanwhile, rats have periodontal tissues that resemble those of humans and have high bone transformation efficiency, making them ideal for studies on tissue remodeling^30^. Based on the methods of Widmer^31^ and Yabushita^32^, different occlusal patterns were established by modifying the occlusal surface of LM1 using adhesive resin, reducing the abrasion of the UM1 in comparison to metal crownsor inlays.

The occlusal contact patterns could influence the flexibility of the dynamic movement and the transfer of occlusal forces into the supporting tissues of the teeth. The intercuspation contacting relationship in the OA group allowed the occlusalforces to be dispersed perpendicular to the slope of the cusp and transferred along the long axis of the tooth, while the OI group with contact interferences generated horizontal or lateral forces when occluding. The OD group without occlusal contact prevented the transmission of forces generated by masticatory muscle contraction through the interactive contact of the maxillary and mandibular teeth into the supporting tissues. Teeth in the OI group showed the most relapse distance. Incorrect occlusal contact generated fractional forces in the horizontal direction that displace the teeth, affecting the stability and leading to undesirable changes^4^. The teeth in the OA group exhibited the most stability after active OTM, indicating that the smooth and adequate intercuspation contacts of molars helped resist the stress generated in the fibers and maintain tooth position stability.

The biological response that occurs in the periodontal tissue during relapse is similar to that during OTM, with active bone remodeling eventually leading to tooth movement. Unlike OTM, the process of relapse movement is continuous and there is no period of stagnation. According to previous studies, the rate of relapse was highest in the first three days after removal of the force appliance, reaching up to 89.9% on day 4, after which the rate of relapse decreased, which was consistent with our result in the relapse rate^33^.

Body weight is regarded as an important indicator of the nutritional status of animals. After the formation of occlusal interference, the OI group exhibited a significant decrease in food intake, higher chewing frequency, and irritable behavioral responses such as face grooming^34^. The decrease in body weight was attributed to the change in chewing mode and the difficulty in contacting the upper and lower incisors with interference in the molar region. The increase in body weight in the OD group was slower when compared to the C and OA groups, given that the absence of contact between the molars prevented the normal exercise of the food grinding function and reduced the chewing efficiency, thereby affecting the nutritional intake and body weight gain.

According to previous studies, IL-1β and IL-6 are important pro-inflammatory factors in response to OTM^35^. Il-1β is a potent stimulator of bone resorption that plays a central role in periodontal tissue remodeling during the early phases of tooth movement and is an early promoter of osteoclasts.IL-6 is able to promote the development of bone resorption by increasing the number of osteoclast precursors or by binding to specific receptors on the osteoclast membrane^36^. Several studies have confirmed the close relationship between IL-6 levels and bone resorption during OTM^37–39^. The effect of different occlusal patterns on IL-1β and IL-6 levels in GCF during relapse was examined in this study. The levels of IL-1β and IL-6 tended to decrease after the removal of the force appliances, suggesting that the presence of the cervical appliance and mechanical force stimulation may have increased the inflammatory response^40^. The results indicated that occlusal interference increased the inflammatory response of the periodontal tissues in the OI group. Meanwhile, the OA group exhibited the lowest levels of IL-1β and IL-6. The maintenance of periodontal tissues stability and the formation of new bone are fundamental for ensuring the teeth retain their newly acquired positions following OTM. The correct transmission of forces through occlusal contacts facilitates the control of local inflammatory responses and prevents bone loss or tooth relapse^41^.

After the orthodontic force device is removed, the periodontal tissue of the moved teeth undergoes adaptive modifications, which mainly involve reduced tension in the periodontal membrane fibers, improved alignment direction, and modification of alveolar bone^42^. This study found that the in the OA group with adequate occlusal contacts, periodontal tissues on the original tension side were more orderly and denser, with a higher volume of collagen fibers compared to groups without adequate occlusal contacts. It was considered that the periodontal fibers adapted to the new position of the teeth due to the occlusal force of the cusp-fossa intercuspation resisting the relapse force generated by fiber retraction^43^.Conversely, occlusal interference accompanied by uneven contraction of periodontal membrane fibers disturbed the alignment of periodontal fibers, resulting in reduced collagen fiber content and instability of teeth^44^. The decrease in collagen fiber content in periodontal tissues suggested that the teeth were more prone to movement and less stable^45^.

Appropriate mechanical force stimulation is significant for bone formation, maintenance, and modification^16^. Lack of proper occlusal force stimulation may lead to bone loss in the alveolar bone^46^. Adequate occlusal contact allows the transfer of occlusal forces along the long axis of the dentition to the roots, the periodontium, and the alveolar bone tissue between arches. In the absence of occlusal contact or in the presence of occlusal interference, occlusal forces are difficult to transfer uniformly, and tooth-supporting tissues may locally lack force stimulation or undergo excessive dental forces. Consequently, pathological changes in the periodontal tissue, such as narrowing of the periodontal membrane, vascular constriction, and deformation of mechanoreceptor structures, may cause bone loss^47,48^. The current study found that the number of osteoclasts was the lowest in the intercuspation contact group and the highest in the group with occlusal interferences. According to some research, during the first three days of relapse, the number of osteoclasts on both sides of the root decreased due to apoptosis or decreased vascular density caused by the removal of exogenous mechanical stress^49^. After seven days of relapsing tooth movement, the number of osteoclasts redistributed and eventually concentrated in direction of relapse on the distal side of the root.

BMP-2, Runx2, OPG, and RANKL play important roles in regulating alveolar bone remodeling during tooth relapse, with BMP-2 and Runx2 promoting osteogenesis while OPG, and RANKL regulating osteolysis^50–53^. The mechanical stress transmission and distribution caused by different occlusal contact patterns also affect the expression of these genes. In this study, teeth with intercuspation contact had dominant osteogenic activity and low osteolytic activity in the new position, which helped to maintain stability in the new position. And the conversion of bone helped to resist the traction force generated by fiber retraction, leading to less relapse. In contrast, teeth with interferences demonstrated active osteolytic activity and unstable positions, predisposing them to relapse. Occlusal trauma from interferences increased RANKL expression and osteoclasticresorptive activity, consistent with the results of previous studies^54^. There was no significant difference in bone remodeling-related gene expression between the OD group and the C group. However, the relapse rate was higher in the group without occlusal contact, given that teeth relapse occurred rapidly in the absence of appropriate occlusal force-stimulated osteogenesis. Liu et al.^55^ found that osteogenic gene expression was downregulated and accompanied by a decrease in bone density when the mandible lacked appropriate occlusal force stimulation.

As the primary muscle exercising function in ascending the mandibular, the masseter plays a crucial role in maintaining the position of mandible.Different occlusal contact patterns can result in adaptive changes in mitochondrial content, muscle fiber cross-sectional area, muscle fiber composition ratio, muscle size, etc.^56^.The results of the study demonstrated no inflammatory microstructural changes in the masseter tissues following experimental tooth movement. However, previous studies found that tooth wear and movement cause injury-like changes in occlusal muscle tissue limited to ultrastructural changes, such as changes in mitochondrial morphology and characteristic structures due to increased Ca2 + content in mitochondria of muscle cells. However, the occlusal interferences caused deteriorating changes in the masseter tissue, leading to overstretched occlusal fibers after dynamic occlusal trauma.

The completion of the masticatory cycle is inextricably linked to the biomechanical function of the TMJ^54^. Previous animal studies investigating the effects of occlusion on histological alterations of the joint have shown that occlusal changes in molar teeth may lead to changes in the TMJ cartilage. These changes include thickening of cartilage tissue, an increase in cartilage tissue volume, changes in chondrocyte morphology and viability^55^, reduction in chondrocyte proliferation and extracellular matrix^56^, increased infiltration of monocytes in cartilage tissue, and chondrogenesis, ossification, and neovascularization of the posterior condyle^57,58^ Physiological alterations of the TMJ contribute to adapting to different forms of occlusal contact and the biological forces generated in the movement of the jaw. However, the long-term presence of non-physiological stimuli may induce pathological changes in the condylar cartilage, producing organic arthropathy and osteoarthritis. A series of studies by Wang et al. depicted that different occlusal patterns resulting from different types of molar movements produced different biological effects in the TMJ. When the maxillary and mandibular teeth were in cusp-fossa intercuspationocclusal relationships, the general morphology and histology of the joint behaved normally. Nevertheless, several studies identified different degrees of degenerative changes in the joint and proliferative alterations in the cartilage when the intercuspationocclusal relationship between arches was lost^54^. In the present study, significant local manifestations of cartilage degeneration were observed. These results may due to the alteration of the mechanical environment in the internal structure of the TMJ caused by the presence of occlusal interference^59^. Expression of RANKL during the remodeling of the periodontal fiber had been studied using various methods in the processes of tooth movement and hyper occlusion^51,60^. Kim et al.^60^ reported the first molars in the control group were laterally expanded by a continuous orthodontic force. Immunostaining for RANKL was performed in horizontal sections of rat periodontium between the bi-furcation level and the middle of the first molar mesiobuccal root. Another study found increased RANKL expression as a result of traumatic occlusion in immunostained sections of mice^51^. At this point, we do not have the necessary toolset to study the localization of RANKL, and the period of sampling would be too long. This study is beyond the scope of this report which focuses on answering the critical questions regarding the influence of occlusion contact on the stability of tooth after OTM itself. The mechanism of tooth movement and the localization of related factors including BMP-2, RANKL, and so on would be further studied in the subsequent experiments.Andthe long-term effects of different occlusion patterns on joint tissues and the effect of occlusion on the human condyle still need to be further investigated.

## Conclusions

In conclusion, the intercuspationocclusal contact pattern without any interference after orthodontic treatment exerted good effects in terms of reducing the inflammatory response of periodontal tissues, promoting the expression of osteogenic genes, and inhibiting the RANKL/OPG pathway, which is conducive to the reduction of relapse movement and stability of tooth after orthodontic movement. However, the absence of occlusal contact can make the position of teeth after OTM unstable, since periodontal tissues could not receive adequate simulation from the occlusal force. Conversely, the presence of occlusal interference may promote the inflammatory response of periodontal tissue, activate the RANKL/OPG pathway, initiate active bone resorption, and accelerate the relapse of orthodontic teeth, accompanied by the pathological alteration of masticatory muscles and joint structures. Therefore, necessary occlusal adjustment after active OTM should be performed to achieve the desired intercuspation contact relationship and adequate contact between arches. It is also essential to eliminate occlusal interferences to achieve stability and a healthy condition of the oromandibular system.

## Data Availability

The datasets generated and analyzed during the current study are available from the corresponding author on reasonable request.
